# Association of Renal Impairment Severity with Surgical Outcomes in Patients with Infective Endocarditis

**DOI:** 10.2174/011573403X353597250515051547

**Published:** 2025-06-03

**Authors:** Jing Yong Ng, Eu Fon Tan, Soubhagyashree Roy, Sungha Cho, Takakazu Ryan Yatoji Tan, Marsioleda Kemberi, Wael I. Awad

**Affiliations:** 1 Barts Heart Centre, St Bartholomew’s Hospital, London, UK;; 2 Barts and The London School of Medicine and Dentistry, Queen Mary University of London, London, UK;; 3 William Harvey Research Institute, Queen Mary University of London, London, UK;; 4 University of South Wales, Cardiff, UK

**Keywords:** Renal impairment, dialysis, endocarditis, valvular surgery, cardiac surgery, atrial fibrillation

## Abstract

**Introduction:**

This study aimed to assess the association of renal impairment (RI) severity on short and mid-term outcomes in patients undergoing cardiac surgery for infective endocarditis (IE).

**Methods:**

Patients undergoing cardiac surgery for IE between January 2010 and October 2022 were included. They were stratified based on preoperative renal function into four groups: Normal (N: Creatinine clearance (CrCl) >85 mL/min), moderate RI (M: CrCl 51-85 mL/min), severe RI (S: CrCl ≤50 mL/min), and haemodialysis-dependent (H). Each group was compared with group N. Survival analysis was performed using Kaplan-Meier curves.

**Results and Discussion:**

A total of 487 patients (N: 198; M: 154; S: 96; H: 39) were included. Mean age 55.92 ± 14.60 years, 375 (77%) males. Groups M, S, and H *vs* N demonstrated more atrial fibrillation [17 (11.0%), 20 (20.8%), 6 (15.4%) *vs* 8 (4.0%); *p<*0.05]. Groups S and H *vs.* N had increased incidence of left ventricular ejection fraction <50% [43 (44.8%), 22 (56.4%) *vs* 43 (21.7%); *p<*0.001] and preoperative cardiogenic shock [16 (16.7%), 13 (33.3%) *vs* 9 (4.5%); *p<*0.001]. The need for postoperative haemodialysis was 21 (13.6%) in M and 23 (23.0%) in S *vs.* 13 (6.6%) in N (*p<*0.05). In-hospital mortality was 13 (8.4%), 21 (21.9%), and 11 (28.2%) *vs.* 12 (6.1%) (*p=*0.388, <0.001, <0.001), and mortality at a mean of 69.1months was 49 (31.8%), 46 (46.9%), 30 (76.9%) *vs.* 49 (24.7%) (*p=*0.142, <0.001, <0.001) in groups M, S, H *vs.* N, respectively.

**Conclusions:**

The incidence of renal impairment in patients with IE undergoing surgery remains high. Early and mid-term outcomes of those with severe RI and haemodialysis dependence are significantly worse.

## 1 INTRODUCTION

Infective endocarditis (IE) is a severe and potentially fatal microbial infection of the inner lining of the heart, most commonly affecting heart valves. In the United Kingdom, IE is relatively uncommon, with an estimated incidence of 10 cases per 100,000 population per year [1]. The primary treatment for infective endocarditis (IE) is antimicrobial therapy; however, surgical intervention is often necessary. It is estimated that 20-25% of patients with IE require valvular surgery, with an in-hospital mortality rate of up to 25% [2-4]. This population of patients also often presents with multiple co-morbidities, including diabetes, renal impairment, and underlying heart conditions [5, 6]. A recent study found that 52.7% of patients with IE had renal dysfunction, highlighting the need to understand better the impact of renal impairment on postoperative outcomes in patients with IE [7]. Preoperative renal impairment is widely recognised as a significant risk factor for increased mortality rates post-cardiac surgery. However, there is a noticeable lack of evidence that specifically explores the postoperative outcomes after valvular surgery among patients with IE and associated renal impairment. [8-10] As such, this study aims to explore the association between different stages of renal impairment and outcomes of valvular heart surgery for patients with IE. The results of such research may inform clinical decision-making.

## 2 MATERIALS AND METHODS

This was a retrospective observational study aimed at investigating the impact of the severity of renal impairment on the outcomes of patients who underwent valvular surgery for infective endocarditis. The study was conducted at a single tertiary centre, and all patients who underwent cardiac surgery between January 2010 and October 2022 were investigated. These patients’ preoperative, perioperative, and postoperative data were retrieved from our clinical database. The data collected included demographic information, medical history, laboratory results, surgical details, and postoperative outcomes, including length of stay, complications, and mortality. The creatinine clearance (CrCl) of all patients was calculated with the Cockcroft-Gault Equation, using the preoperative creatinine level. The patients were then stratified into 4 groups based on their preoperative renal function: Normal renal function (N: CrCl >85 mL/min), moderate renal impairment (M: CrCl 51-85 mL/min), severe renal impairment (S: CrCl ≤50 mL), and haemodialysis-dependent (H).

The primary endpoints of this study were in-hospital mortality and mid-term survival at a follow-up date on 30^th^ September 2023. In-hospital mortality was defined as any death that occurred during the hospitalisation in which the valve surgery was performed, and the status of patients at follow-up was determined using the National Summary Care Record. In addition to short and mid-term mortality, other endpoints that were included in the study were incidence of new-onset haemodialysis, deep sternal wound infections (infection of the surgical site within 30 days of surgery), new cerebral vascular accidents (CVAs), and postoperative length of hospital stay. The other variables collected included comorbidities peripheral vascular disease (PVD), diabetes, hypertension, atrial fibrillation, and chronic obstructive pulmonary disease (COPD). Operative urgency was classified into elective, urgent, and emergency/salvage. Urgent operations were defined as those performed during the same hospital admission, whereas emergency/salvage were those performed within 24 hours of presentation.

Continuous data was presented as mean ± 1 standard deviation (SD), and categorical data was presented as frequency (%). The normality of continuous variables was assessed using the Shapiro-Wilk test, which revealed that all continuous data was not normally distributed. Hence, differences among these groups were tested with the Kurskal-Wallis test. Categorical variables were analysed using the chi-squared test, and Yates’ correction was used for data with expected values less than 5 to prevent overestimation of p values. To investigate the differences between groups, additional statistical analyses were performed using the Mann-Whitney U test and chi-squared test to compare each group (M, S, and H) to the normal (N) group. The result was considered statistically significant if the p-value was less than 0.05.

Survival analysis for each of the renal impairment groups was performed and illustrated using a Kaplan-Meier curve, and log-rank tests were used to determine if differences were significant. The 1-, 3-, and 5-year survival rates and their respective 95% confidence intervals were calculated using the Greenwood formula.

## 3 RESULTS

**Fig. (1):**
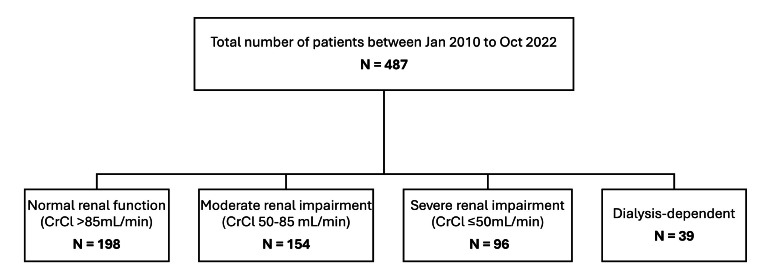
This figure illustrates the classification of 487 patients who underwent surgery for infective endocarditis, categorised based on preoperative renal function. CrCl: Creatinine clearance, calculated from preoperative creatinine levels using the Cockcroft–Gault equation.

From January 2010 to October 2022, a total of 487 patients underwent cardiac surgery due to infective endocarditis. Among these cases, 289 (59.3%) patients had renal impairment, with 154 (31.6%) having moderate renal impairment, 96 (19.7%) having severe renal impairment, and 39 (8%) were haemodialysis-dependent before the surgical procedure (Fig. **1**).

Table **1** shows the preoperative characteristics for all patients. Patient profiles in each renal impairment group were compared to the normal group. Patients with normal renal function had a mean age of 48.17 ± 13.4 years, while patients with renal impairment were older [M: 60.6 ± 12.8 years, S: 64.6 ± 13.1 years, and H: 55.7 ± 10.7 years (*p<*0.001, <0.001, 0.032)]. Preoperatively, 103 (66.9%) with moderate renal dysfunction, 76 (79.2%) with severe renal impairment, and 35 (89.7%) receiving haemodialysis reported New York Heart Association (NYHA) Class III/IV symptoms, *versus* 117 (59.1%) of those with normal renal function (*p=*0.134, <0.001, <0.001 respectively). The findings for the Canadian Cardiovascular Society (CCS) class were similar. At presentation, 15 (9.7%), 13 (13.5%), and 7 (17.9%) patients in groups M, H, and S reported CCS class III/IV symptoms, compared to 9 (4.5%) in group N (*p=* 0.055, 0.006, 0.007).

As expected, patients with impaired renal function also displayed a heightened cardiovascular risk profile. Specifically, 90 (58.4%) in group M, 66 (68.8%) in group S, and 23 (59.0%) in group H were hypertensive, a significantly higher prevalence compared to 65 (32.8%) among those with normal renal function. A higher prevalence of atrial fibrillation among patients with renal insufficiency was also noted, with 17 (11.0%) in group M, 20 (20.8%) in group S, and 6 (15.4%) in group H, compared to 8 (4.0%) in group N (*p=*0.011, <0.001, 0.018 respectively). In addition, diabetes mellitus was also more common among patients with renal dysfunction [M: 29 (18.8%), S: 20 (20.8%), H: 7 (17.9%), *vs.* N: 16 (8.1%); *p=*0.003, 0.002, 0.108. The prevalence of peripheral vascular disease (PVD) was also noted to be higher among patients with renal dysfunction [M: 7 (4.5%), S: 10 (10.4%), H: 6 (15.4%), *vs* N: 1 (0.5%); *p=*0.031, <0.001, <0.00^1^].

Table **2** shows the operative details of all patients. Patients within groups S and H were more likely to have undergone emergency/salvage operations compared to those from group N [S: 32 (33.3%), H: 25 (64.1%) *vs* N: 39 (19.7%); *p=*0.010, <0.00^1^]. Moreover, patients with renal impairment exhibited increasingly higher mean EuroSCORE II (European System for Cardiac Operative Risk Evaluation II) values in comparison to those with normal renal function (M: 10.76, S: 24.73, H: 30.25, N: 7.81; all *p<*0.001). Among the H group, 4 (10.3%) required intra-operative intra-aortic balloon pump counterpulsation to aid weaning from cardiopulmonary bypass (CPB), compared to 1 (0.5%) patient among those with normal renal function (*p=*0.001). Group H patients also had a higher incidence of tracheostomy *versus* group N [3 (17.6% *vs* 4 (2%); *p=*0.034).

Patients with renal impairment were more likely to receive a bioprosthetic rather than a mechanical valve. Thus, in patients with moderate renal impairment, 92 (59.7%) received bioprosthetic valves, increasing to 71 (74.0%) for patients with severe renal impairment and to 32 (82.1%) in those receiving haemodialysis. In contrast, among patients with normal renal function, 74 (37.4%) received bioprosthetic valves.

Table **3** shows the surgical outcomes of all patients. Patients with moderate and severe renal dysfunction had a greater need for new-postoperative haemodialysis compared to those without renal impairment [M: 21 (13.6%), S: 23 (24.0%) *vs.* N: 13 (6.6%); both *p<*0.00^1^]. Higher incidence of re-sternotomy for bleeding/tamponade was also observed in group S compared to N [11 (11.5%) *vs* 9 (4.5%), respectively; *p=*0.0^27^].

Patients with renal impairment had a higher in-hospital mortality rate. The in-hospital mortality rates for patients with varying degrees of renal impairment were as follows: M: 13 (8.4%), S: 21 (21.9%), and H: 11 (28.2%), *versus* 12 (6.1%) observed in group N (*p=*0.388, <0.001, <0.001).

The overall survival rate at discharge was 88.3% [N: 187 (94.4%), M: 141 (91.6%), S: 75 (78.1%), and H: 28 (71.8%)]. A total of 8 patients (N: 1, M: 3, S: 4) were lost at follow-up. These individuals were international patients who had been transferred to our centre for surgery, and their status could not be accessed through the National Summary Care Record database after discharge. At a mean follow-up of 69.1 months, the survival rates were significantly lower among patients with severe renal impairment and those with preoperatory haemodialysis. The number of patients who were alive at a mean follow-up of 69.1 months was 50 (52.1%) and 9 (23.1%) for group S and H, respectively, *versus* group N, with a survival rate of 149 (75.3%) at follow-up.

**Fig. (2):**
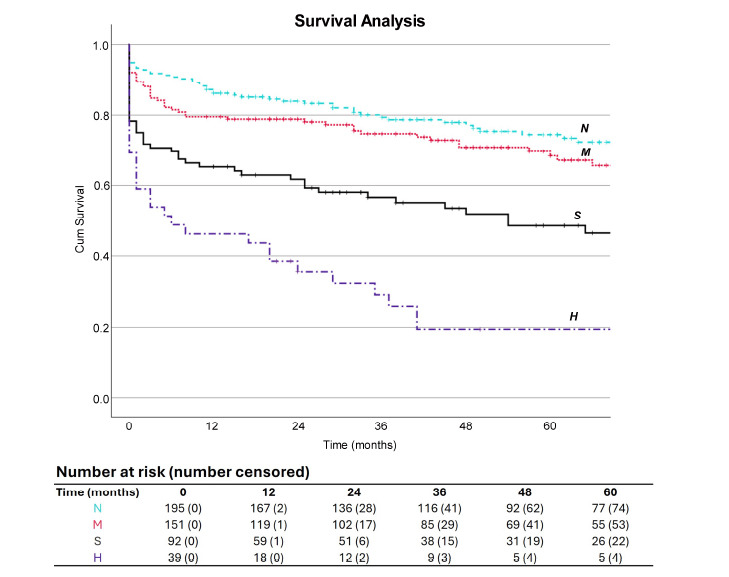
Kaplan-Meier Survival curves, stratified by preoperative renal function (log-rank Mantel-Cox *p<*0.001). Pairwise analysis revealed significant differences for normal-haemodialysis (*p<*0.001), normal-severe (*p=*0.002), moderate-haemodialysis (*p=*0.002), moderate-severe (*p=*0.004), and severe-haemodialysis (*p=*.002). **Abbreviations:** N: normal, M: moderate renal impairment, S: severe renal impairment, H: haemodialysis-dependence.

The Kaplan-Meier survival curves (Fig. **2**) revealed a significant difference between the groups (log-rank Mantel-Cox *p<*0.001). Pairwise comparisons unveiled significant distinctions among the following pairs: normal-severe, normal-haemodialysis, moderate-severe, moderate-haemo-dialysis, and severe-haemodialysis (all *p<*0.05). No significant difference in survival outcomes was observed in the normal-moderate pair. The median survival for groups S and H were estimated to be 54 and 6 months, respectively. The 1-, 3-, and 5-year survival rates are displayed in Table **4**.

Further subgroup analysis revealed that there was no significant difference in in-hospital mortality between those who had valvular surgery with and without CABG [valve: 48 (12.5%) *vs.* valve + CABG: 9 (17.0%), *p=*0.305] (Table **S1**).

Preoperative cardiogenic shock was also significantly more common in groups S and H compared to N [M: 12 (7.8%), S: 16 (16.7%), H: 13 (33.3%) *vs* N: 9 (4.5%); *p=*0.202, <0.001, <0.001 respectively]. Higher mortality rates were observed among patients who had preoperative cardiogenic shock, with an in-hospital mortality of 32%, as compared to 9.4% for those without (*p<*0.001) (Table **S2**).

Subgroup analysis of patients with atrial fibrillation *versus* those in normal sinus rhythm is shown in Table **S3**. Among the 51 patients who had atrial fibrillation preoperatively, the in-hospital mortality rate was 11 (21.6%), compared to 44 (10.1%) in those in sinus rhythm (*p=*.014).

## 4 DISCUSSION

Preoperative renal dysfunction is a well-recognized independent predictor of mortality and morbidity following cardiac surgery. However, there are limited studies specifically exploring the association of renal impairment severity on the outcomes of cardiac surgery in patients with infective endocarditis [11]. This study represents a single-centre 12-year surgical experience in patients who underwent cardiac surgery for infective endocarditis. At the time of surgery, 59.3% of patients in our cohort demonstrated varying degrees of renal dysfunction. We showed that preoperative severe renal impairment and haemodialysis dependence were associated with worse clinical outcomes, including increased in-hospital and mid-term mortality. Additionally, patients with severe renal impairment experienced a higher incidence of complications, such as re-sternotomy for bleeding or tamponade, and an increased need for new haemodialysis postoperatively. Patients with moderate renal impairment had similar postoperative outcomes, including mortality, compared to those with normal renal function.

Our study is the first to investigate the association between varying degrees of renal impairment and the outcomes of patients undergoing cardiac surgery for infective endocarditis. The in-hospital mortality rate for patients with moderate renal impairment was shown to be 8.4%, which was comparable to those with normal renal function (6.1%). Patients with severe renal dysfunction and those who required haemodialysis, however, demonstrated significantly higher in-hospital mortality rates of 21.9% and 28.2%, respectively. Our findings align consistently with prior research on the outcomes of cardiac surgery for infective endocarditis in dialysis patients. In a recent study by Liau *et al*. involving 116 dialysis-treated patients, an in-hospital mortality rate of 43.1% was reported [12]. Similarly, in another investigation by Guo *et al*., which encompassed 125 dialysis-treated patients undergoing surgery for infective endocarditis, an in-hospital mortality rate of 26% was observed [13].

The Kaplan-Meier survival analysis revealed a discernible trend in mid-term survival rates. Patients with normal renal function and those with moderate renal impairment exhibited similar survival rates throughout the follow-up period. In contrast, the cohort with severe renal dysfunction experienced a notable decline in survival outcomes, which became even more pronounced in individuals who were haemodialysis-dependent preoperatively. In our study, the overall 5-year survival rate was 19.4% for the haemodialysis-dependent group. Raza *et al*. and Guo *et al*. also investigated long-term survival in dialysis patients undergoing surgery for IE, reporting 5-year survival rates of 24% and 26.4%, respectively [10, 13].

Dhanani *et al*. looked at the impact of preoperative renal function on cardiac surgery in general (CABG, valvular, thoracic aortic surgery, or combined). Their survival analysis showed similar long-term outcomes in patients with eGFR (estimated glomerular filtration rate) of >90 mL/min and 60-90mL/min. The survival rates then progressively worsened in the 30-59 mL/min, <30 mL/min, and dialysis-dependent groups [14]. A similar trend was also seen in a major study by Cooper *et al*. that investigated the impact of renal dysfunction on the outcomes of coronary bypass graft surgery. They revealed that of the 483,914 patients, those with glomerular filtration rate (GFR) between 30 to 59 mL/min had 55% higher odds for operative mortality in comparison to the normal group (GFR ≥90). The number rose to over 3.8 times higher among the dialysis-dependent population. In their study, patients with mild renal dysfunction (GFR 60-89) also had operative mortality that was similar to that of the normal group [15].

The considerably worse survival rates observed in patients with severe renal impairment and those requiring preoperatory haemodialysis often prompt clinicians to question whether surgery is worthwhile in this group of patients. However, prior research consistently affirms that surgical intervention for patients with IE, including those who were dialysis-dependent, is associated with substantially improved outcomes compared to those treated medically [10, 16, 17].

The association between renal impairment and worse outcomes post-surgery for IE is a complex issue, as several factors are likely involved in the observed correlation. One key factor is the progressive deterioration of renal function prior to surgery, which can be further impacted by the choice of medical management and the increased number of comorbidities in this population. Patients with IE are often treated with medications such as diuretics and aminoglycosides. These medications are known for their nephrotoxicity, exacerbating renal impairment. The increased number of comorbidities in these populations is another well-recognised factor that predisposes patients to an elevated risk of postoperative complications and negatively impacts long-term morbidity and mortality [18, 19]. This relationship was reflected in our study where those with severe renal impairment and haemodialysis-dependent patients were not only older but also displayed a higher prevalence of various comorbidities including higher incidence of left ventricular dysfunction (LVEF <50%), NYHA class III/IV symptoms, diabetes, peripheral vascular disease, and atrial fibrillation. A higher proportion of these patients also developed cardiogenic shock preoperatively.

In our study, the prevalence of atrial fibrillation was higher among patients with impaired renal function, and those with atrial fibrillation exhibited higher in-hospital mortality rates of 21.6% *versus* 10.1% in patients with normal sinus rhythm. The association between chronic kidney disease (CKD) and atrial fibrillation (AF) has been previously reported, with an increased risk of AF as renal function declines. The exact pathophysiology remains unclear, but several potential mechanisms have been suggested, including chronic inflammation, overactivation of the renin-angiotensin-aldosterone system, electrolyte imbalance, chronic anaemia, and accumulation of toxins [20, 21]. Preoperative atrial fibrillation may have also contributed to the observed poorer clinical outcomes. Atrial fibrillation is known to worsen the postoperative haemodynamic function of the heart, resulting in a higher risk of complications and prolonged stay in the intensive care unit [22]. In a study that involved more than 1000 cardiac surgery patients, Kim *et al*. showed that irrespective of procedure types, preoperative AF exposed patients to a higher cumulative risk of overall mortality and was associated with poorer long-term outcomes [23].

The ongoing controversies about valve selection for patients with renal impairment highlight the need for further exploration, especially as the ageing patient population increasingly requires valve replacement surgery. Early concerns about the durability of bioprosthetic valves, particularly with evidence of accelerated valve calcification and degeneration in patients with renal failure, prompted the 1998 AHA/ACC guidelines to favour mechanical valves in such cases [24]. Our study indicated that the majority of patients in both severe renal impairment and haemodialysis groups had bioprosthetic valves (74.0% and 82.1%, respectively). To date, no studies have specifically compared bioprosthetic and mechanical valves among renal-impaired patients undergoing surgery for IE. Recent studies on dialysis patients undergoing valve replacement for all indications, however, report no significant differences in postoperative complications and survival [24-26]. Kim *et al*. conducted a meta-analysis involving 9,857 dialysis patients and found that reoperation rates at a 3.6-year follow-up were similar between those receiving bioprosthetic and mechanical valves [27]. Chan *et al*. also revealed similar rates of 30-day hospital readmission (bioprosthetic 35.2% *vs.* mechanical 36.4%, *p=*0.99) and readmission for bleeding (bioprosthetic 3.7% *vs.* mechanical 3.2%, *p=*0.89) between the two groups [24]. In their study, a total of 30 (30.9%) patients underwent valve replacement due to infective endocarditis, and none required reoperations at 5 years.

Several studies have emphasised the limited long-term survival of dialysis patients, many of whom are unlikely to outlive the lifespan of their bioprosthetic valves or require reoperation due to valve degeneration. Guo *et al*.’s study supports this with a very low rate of reoperation in their dialysis group, with five- and ten-year incidences of 0% and 2.0%, respectively [13]. The recent guideline revisions, exemplified by the European Society of Cardiology (ESC), now offer recommendations for valve selection based on patient-specific factors, including age, life expectancy, lifestyle, comorbidities, surgical considerations (*e.g*., bleeding and thromboembolic risks), and patient preferences [26-28].

Optimising preoperative renal function, preventing iatrogenic renal impairment, or considering earlier surgical intervention before the decline of renal function may influence the outcomes in patients with infective endocarditis complicated by renal insufficiency. Further studies should be directed towards exploring strategies to enhance preoperative renal function in this specific patient population and exploring the subsequent effects on outcomes and postoperative complications.

## 5 LIMITATIONS

Our study has several limitations that warrant consideration. Firstly, being retrospective, our study may have been vulnerable to selection bias and other confounding factors. Echocardiographic findings such as vegetation sizes and the presence of paravalvular involvement were also unavailable in the study, resulting in difficulty in assessing disease severity. Another limitation of our study is the lack of data on nephrotoxic medications the patients received, such as diuretics, aminoglycosides, and contrast iodine, which may worsen renal impairment in infective endocarditis patients. As a result, we could not assess their impact on outcomes. We also acknowledge our study may be limited by sample size, particularly in the haemodialysis group, potentially affecting statistical power. However, despite these constraints, observed differences in outcomes remain statistically significant and clinically relevant. Moreover, as this analysis was conducted at a single centre, its reproducibility and generalizability may be limited compared to the broader scope and diversity that a multi-centre study could offer.

## CONCLUSION

Renal impairment remains highly prevalent in patients undergoing surgery for infective endocarditis (IE) and significantly impacts early and mid-term outcomes. Our study reveals that severe renal impairment and haemodialysis dependence were associated with higher in-hospital mortality and worse 5-year survival compared to those with normal renal function. These patients also had a higher incidence of postoperative complications, including the need for new haemodialysis and re-sternotomy for bleeding. In contrast, moderate renal impairment did not significantly affect mortality or survival. Given these findings, optimising renal function preoperatively and implementing renal protective strategies perioperatively may help improve outcomes in this high-risk group.

## Figures and Tables

**Table 1:** Preoperative patient characteristics.

**Variable**	**ALL**	**Normal**	**Moderate**	**Severe**	**Haemodialysis**	***P* values**		
	**N=487**	**N=198**	**N=154**	**N=96**	**N=39**			
	**n/487 (%)**	**n/198 (%)**	**n/154 (%)**	**n/96 (%)**	**n/39 (%)**	**M *vs* N**	**S *vs* N**	**H *vs* N**
Male sex	375 (77.0%)	149 (75.3%)	128 (83.1%)	66 (68.8%)	32 (82.1%)	0.074	0.238	0.361
Mean Age (years)	55.92 ± 14.60	48.17 ± 13.43	60.60 ± 12.76	64.59 ± 13.11	55.66 ± 10.69	<0.001	<0.001	0.032
BMI	26.18 ± 6.27	26.28 ± 7.07	26.01 ± 5.81	26.04 ± 5.62	26.66 ± 5.40	0.103	0.730	0.802
Mean pre-op length of stay	7.73 ± 9.71	8.03 ± 9.62	7.25 ± 10.25	7.17 ± 8.92	9.48 ± 9.89	0.384	0.564	0.654
NYHA Class III/IV	331 (68.0%)	117 (59.1%)	103 (66.9%)	76 (79.2%)	35 (89.7%)	0.134	<0.001	<0.001
No chest pain	337 (69.2%)	144 (72.7%)	102 (66.2%)	66 (68.8%)	25 (64.1%)	0.188	0.479	0.276
CCS Class III/IV	44 (9.0%)	9 (4.5%)	15 (9.7%)	13 (13.5%)	7 (17.9%)	0.055	0.006	0.007
LVEF <50%	150 (30.8%)	43 (21.7%)	42 (27.3%)	43 (44.8%)	22 (56.4%)	0.227	<0.001	<0.001
Pre-op atrial fibrillation	51 (10.5%)	8 (4.0%)	17 (11.0%)	20 (20.8%)	6 (15.4%)	0.011	<0.001	0.018
Previous Stroke/TIA	104 (21.4%)	39 (19.7%)	37 (24.0%)	17 (17.7%)	11 (28.2%)	0.327	0.684	0.234
PVD	24 (4.9%)	1 (0.5%)	7 (4.5%)	10 (10.4%)	6 (15.4%)	0.031	<0.001	<0.001
COPD	41 (8.4%)	13 (6.6%)	17 (11.0%)	8 (8.3%)	3 (7.7%)	0.136	0.581	0.926
Hypertension	244 (50.1%)	65 (32.8%)	90 (58.4%)	66 (68.8%)	23 (59.0%)	<0.001	<0.001	0.002
Diabetes	72 (14.8%)	16 (8.1%)	29 (18.8%)	20 (20.8%)	7 (17.9%)	0.003	0.002	0.108
Diet/oral therapy	56 (11.5%)	15 (7.6%)	25 (16.2%)	13 (13.5%)	3 (7.7%)	0.011	0.102	0.760
Insulin dependence	16 (3.3%)	1 (0.5%)	4 (2.6%)	7 (7.3%)	4 (10.3%)	0.233	0.003	0.001
Hypercholesterolaemia*	97 (33.6%)	22 (18.6%)	45 (47.4%)	23 (39.7%)	7 (41.2%)	<0.001	0.003	0.034
Smoking status	-	-	-	-	-	-	-	-
Smoker	79 (16.2%)	37 (18.7%)	26 (16.9%)	9 (9.4%)	7 (17.9%)	0.661	0.039	0.914
Ex-smoker (>1 month)	163 (33.5%)	56 (28.3%)	54 (35.1%)	42 (43.8%)	11 (28.2%)	0.173	0.008	0.992
CAD	60 (12.3%)	13 (6.6%)	26 (16.9%)	16 (16.7%)	5 (12.8%)	0.002	0.006	0.309
One/Two vessel disease	42 (8.6%)	7 (3.5%)	20 (13.0%)	10 (10.4%)	5 (12.8%)	<0.001	0.018	0.044
Three vessel disease/LMS	18 (3.7%)	6 (3%)	6 (3.9%)	6 (6.3%)	0	0.847	0.172	0.587
Previous MI	28 (5.7%)	5 (2.5%)	11 (7.1%)	9 (9.4%)	3 (7.7%)	0.039	0.022	0.251
Previous cardiac surgery	88 (18.1%)	28 (14.1%)	28 (18.2%)	25 (26.0%)	7 (17.9%)	0.304	0.013	0.540
Previous valvular surgery	79 (16.2%)	24 (12.1%)	27 (17.5%)	22 (22.9%)	6 (15.4%)	0.152	0.017	0.767
Previous CABG	18 (3.7%)	5 (2.5%)	2 (1.3%)	9 (9.4%)	2 (5.1%)	0.665	0.022	0.719
Previous PCI	24 (4.9%)	5 (2.5%)	2 (1.3%)	12 (12.6%)	5 (12.8%)	0.665	<0.001	0.013

**Note:** *P* values in bold indicate statistical significance. *Data only available for 289 patients. **Abbreviations:** BMI: body mass index, CCS: canadian cardiovascular society, COPD: chronic obstructive pulmonary disease, LMS: left main stem, LVEF: left ventricular ejection fraction, MI: myocardial infarction, NYHA: New York heart association, PVD: peripheral vascular disease, TIA: transient ischaemic stroke.

**Table 2:** Operative details.

**Variable**	**ALL**	**Normal**	**Moderate**	**Severe**	**Haemodialysis**	***P* values**		
	**N=487**	**N=198**	**N=154**	**N=96**	**N=39**			
	**n/487 (%)**	**n/198 (%)**	**n/154 (%)**	**n/96 (%)**	**n/39 (%)**	**M *vs.* N**	**S *vs.* N**	**H *vs.* N**
Operative Urgency	-	-	-	-	-	-	-	-
Urgent	349 (71.7%)	159 (80.3%)	112 (72.7%)	64 (66.7%)	14 (35.9%)	0.094	0.010	<0.001
Emergency/Salvage	138 (28.3%)	39 (19.7%)	42 (27.3%)	32 (33.3%)	25 (64.1%)	0.094	0.010	<0.001
EuroScoreII	13.54 ±16.48	7.81 ± 11.04	10.76 ± 12.30	24.73 ± 20.15	30.25 ± 24.53	<0.001	<0.001	<0.001
Types of procedure	-	-	-	-	-	-	-	-
Valve only	433 (88.9%)	185 (93.4%)	131 (85.1%)	83 (86.5%)	34 (87.2%)	0.010	0.048	0.309
Valve + CABG	54 (11.1%)	13 (6.6%)	23 (14.9%)	13 (13.5%)	5 (12.8%)	0.010	0.048	0.309
Valve operated								
Single valve	366 (75.2%)	157 (79.3%)	109 (70.8%)	70 (72.9%)	30 (76.9%)	0.065	0.222	0.740
Aortic	221 (45.5%)	93 (47.0%)	61 (39.6%)	46 (47.9%)	21 (53.8%)	0.167	0.879	0.432
Mitral	138 (28.3%)	60 (30.3%)	46 (29.9%)	24 (25.0%)	8 (20.5%)	0.930	0.345	0.217
Aortic + Mitral	85 (17.5%)	29 (14.6%)	32 (20.8%)	19 (19.8%)	5 (12.8%)	0.132	0.263	0.766
Others	43 (8.8%)	16 (8.1%)	15 (9.7%)	7 (7.3%)	5 (12.9%)	0.586	0.813	0.520
Valve implanted								
Bioprosthetic valve	268 (54.8%)	74 (37.4%)	92 (59.7%)	71 (74.0%)	32 (82.1%)	<0.001	<0.001	<0.001
Mechanical valve	194 (39.8%)	109 (55.1%)	56 (36.4%)	23 (24.0%)	6 (15.4%)	<0.001	<0.001	<0.001
Valve repair	26 (5.3%)	15 (7.6%)	7 (4.5%)	4 (4.2%)	0 (0.0%)	0.244	0.265	0.157
CPB time (min) ± SD	141.00 ± 75.72	139.65 ± 66.57	139.12 ± 79.04	148.99 ± 89.10	135.19 ± 72.89	0.475	0.694	0.547
Cross clamp time (min) ± SD	104.59 ± 53.69	106.17 ± 49.49	104.95 ± 58.36	103.83 ± 56.21	97.00 ± 50.75	0.413	0.498	0.169
IABP Used	12 (2.5%)	1 (0.5%)	3 (1.9%)	4 (4.2%)	4 (10.3%)	0.447	0.072	0.001

**Abbreviations:** CABG: coronary artery bypass graft, CPB: cardiopulmonary bypass time, IABP: intra-aortic balloon pump. *P* values in bold indicate statistical significance.

**Table 3:** Postoperative outcomes.

**Variable**	**ALL**	**Normal**	**Moderate**	**Severe**	**Haemodialysis**	***P* values**		
	**N=487**	**N=198**	**N=154**	**N=96**	**N=39**			
	**n/487 (%)**	**n/198 (%)**	**n/154 (%)**	**n/96 (%)**	**n/39 (%)**	**M *vs* N**	**S *vs* N**	**H *vs* N**
Mean postoperative stay (days) ± SD	22.3 ± 17	20.2 ± 12.2	22.5 ± 17	23.9 ± 17.6	28.7 ± 31.2	0.453	0.266	0.481
Tracheostomy*	16 (3.3%)	4 (2.0%)	6 (6.3%)	3 (5.1%)	3 (17.6%)	0.516	0.874	0.034
Post-op IABP Used	7 (1.4%)	1 (0.5%)	2 (1.3%)	3 (3.1%)	1 (2.6%)	0.827	0.200	0.743
New haemodialysis	57 (11.7%)	13 (6.6%)	21 (13.6%)	23 (23.0%)	-	0.026	<0.001	-
Re-sternotomy for bleeding/tamponade	29 (6.0%)	9 (4.5%)	7 (4.5%)	11 (11.5%)	2 (5.1%)	1.000	0.027	0.796
Deep sternal wound infection/chest infection	2 (0.4%)	1 (0.5%)	0 (0.0%)	1 (1.0%)	0 (0.0%)	0.900	0.817	0.365
Re-operation for valvular problems	3 (0.6%)	2 (1.0%)	0 (0.0%)	1 (1.0%)	0 (0.0%)	0.592	0.553	0.743
New Stroke	21 (4.3%)	7 (3.5%)	9 (5.8%)	5 (5.2%)	0 (0.0%)	0.302	0.715	0.500
In hospital mortality	57 (11.7%)	12 (6.1%)	13 (8.4%)	21 (21.9%)	11 (28.2%)	0.388	<0.001	<0.001
Mortality at mean follow-up of 69 months	173 (35.5%)	49 (24.7%)	49 (31.8%)	46 (46.9%)	30 (76.9%)	0.142	<0.001	<0.001

**Abbreviations:** IABP: intra-aortic balloon pump. *P* values in bold indicate statistical significance. *Data only available for 289 patients.

**Table 4:** 1-, 3-, and 5-year survival with 95% confidence interval.

**Renal Function Status**	**1-y Survival**		**3-y Survival**		**5-y Survival**	
	**%**	**95% CI**	**%**	**95% CI**	**%**	**95% CI**
Normal (n=197)	86.1	81.3 – 91.0	79.3	73.4 – 85.2	73.4	66.5 – 80.4
Moderate (n=151)	78.8	72.3 – 85.3	74.7	67.6 – 81.8	68.4	60.2 – 76.5
Severe (n=92)	65.9	56.2 – 75.7	57.2	46.8 – 67.6	46.9	35.5 – 58.3
Haemodialysis (n=39)	46.2	30.5 – 61.8	29.0	14.2 – 43.9	19.4	6.0 – 32.7

## Data Availability

The authors confirm that the data supporting the findings of this research are available within the article.

## LIST OF ABBREVIATIONS

COPD: Chronic Obstructive Pulmonary Disease
CrCl: Creatinine Clearance
CVAs: Cerebral Vascular Accidents
IE: Infective Endocarditis
PVD: Peripheral Vascular Disease
RI: Renal Impairment

## STANDARDS OF REPORTING

STROBE guidelines were followed.

## SUPPLEMENTARY MATERIAL

PRISMA checklist and supplementary material are available on the publisher’s website along with the published article.

## References

[1] Yallowitz A.W., Decker L.C. (2022). StatPearls..

[2] Jensen A.D., Østergaard L., Petersen J.K. (2022). Surgical treatment of patients with infective endocarditis: Changes in temporal use, patient characteristics, and mortality—a nationwide study.. BMC Cardiovasc. Disord..

[3] Jault F., Gandjbakhch I., Rama A. (1997). Active native valve endocarditis: Determinants of operative death and late mortality.. Ann. Thorac. Surg..

[4] Castillo J.C., Anguita M.P., Ramírez A. (2000). Long term outcome of infective endocarditis in patients who were not drug addicts: A 10 year study.. Br. Heart J..

[5] Maser M., Freisinger E., Bronstein L. (2021). Frequency, mortality, and predictors of adverse outcomes for endocarditis in patients with congenital heart disease: Results of a nationwide analysis including 2512 endocarditis cases.. J. Clin. Med..

[6] Gopal K., Krishna N., Varma P.K. (2021). Surgery for infective endocarditis: Analysis of factors affecting outcome.. Indian J. Thorac. Cardiovasc. Surg..

[7] Von Tokarski F., Lemaignen A., Portais A. (2020). Risk factors and outcomes of early acute kidney injury in infective endocarditis: A retrospective cohort study.. Int. J. Infect. Dis..

[8] Lok C.E., Austin P.C., Wang H., Tu J.V. (2004). Impact of renal insufficiency on short- and long-term outcomes after cardiac surgery.. Am. Heart J..

[9] Vohra H.A., Armstrong L.A., Modi A., Barlow C.W. (2014). Outcomes following cardiac surgery in patients with preoperative renal dialysis.. Interact. Cardiovasc. Thorac. Surg..

[10] Raza S., Hussain S.T., Rajeswaran J. (2017). Value of surgery for infective endocarditis in dialysis patients.. J. Thorac. Cardiovasc. Surg..

[11] Legrand M., Pirracchio R., Rosa A. (2013). Incidence, risk factors and prediction of post-operative acute kidney injury following cardiac surgery for active infective endocarditis: An observational study.. Crit. Care.

[12] Liau S.K., Kuo G., Chen C.Y. (2021). In-Hospital and long-term outcomes of infective endocarditis in chronic dialysis patients.. Int. J. Gen. Med..

[13] Guo M., St Pierre E., Clemence J. (2021). Impact of chronic renal failure on surgical outcomes in patients with infective endocarditis.. Ann. Thorac. Surg..

[14] Dhanani J., Mullany D.V., Fraser J.F. (2013). Effect of preoperative renal function on long-term survival after cardiac surgery.. J. Thorac. Cardiovasc. Surg..

[15] Cooper W.A., O’Brien S.M., Thourani V.H. (2006). Impact of renal dysfunction on outcomes of coronary artery bypass surgery: Results from the society of thoracic surgeons national adult cardiac database.. Circulation.

[16] Leither M.D., Shroff G.R., Ding S., Gilbertson D.T., Herzog C.A. (2013). Long-term survival of dialysis patients with bacterial endocarditis undergoing valvular replacement surgery in the United States.. Circulation.

[17] Alvarado-Alvarado J.A., Vidal-Morales G., Velázquez-Silva R.I. (2021). Surgical procedure versus medical treatment for infective endocarditis associated to mortality in Mexican population.. Arch. Cardiol. Mex..

[18] Sarnak M.J., Levey A.S., Schoolwerth A.C. (2003). Kidney disease as a risk factor for development of cardiovascular disease.. Hypertension.

[19] Mehta R.H., Grab J.D., O’Brien S.M. (2008). Clinical characteristics and in-hospital outcomes of patients with cardiogenic shock undergoing coronary artery bypass surgery: Insights from the Society of Thoracic Surgeons National Cardiac Database.. Circulation.

[20] Gadde S., Kalluru R., Cherukuri S.P., Chikatimalla R., Dasaradhan T., Koneti J. (2022). Atrial fibrillation in chronic kidney disease: An overview.. Cureus.

[21] Harada M., Nattel S. (2021). Implications of inflammation and fibrosis in atrial fibrillation pathophysiology.. Card. Electrophysiol. Clin..

[22] Banach M., Goch A., Misztal M. (2008). Relation between postoperative mortality and atrial fibrillation before surgical revascularization--3-year follow-up.. Thorac. Cardiovasc. Surg..

[23] Kim H.H., Kim J.H., Lee S. (2022). Long-term outcomes of preoperative atrial fibrillation in cardiac surgery.. J. Chest Surg..

[24] Chan P.G., Sultan I., Gleason T.G., Navid F., Kilic A. (2019). Mechanical versus bioprosthetic valves in patients on dialysis.. J. Thorac. Dis..

[25] Herzog C.A., Ma J.Z., Collins A.J. (2002). Long-term survival of dialysis patients in the United States with prosthetic heart valves: Should ACC/AHA practice guidelines on valve selection be modified?. Circulation.

[26] Kim K.S., Belley-Côté E.P., Gupta S. (2022). Mechanical versus bioprosthetic valves in chronic dialysis: A systematic review and meta-analysis.. Can. J. Surg..

[27] Cetinkaya A., Poggenpohl J., Bramlage K. (2019). Long-term outcome after mitral valve replacement using biological versus mechanical valves.. J. Cardiothorac. Surg..

[28] Vahanian A., Beyersdorf F., Praz F. (2022). 2021 ESC/EACTS Guidelines for the management of valvular heart disease.. Eur. Heart J..

