# Supplementary material for: Association of Renal Impairment Severity with Surgical Outcomes in Patients with Infective Endocarditis
Source: Curr Cardiol Rev. 2025 Jun 3;22(1):E1573403X353597. doi: 10.2174/011573403X353597250515051547 (PMC12951051; doi:10.2174/011573403X353597250515051547)
Supplement: Supplementary file 1 [file CCR-22-1-E1573403X353597_SD1.pdf]

# Supplementary Material

# Association of Renal Impairment Severity with Surgical Outcomes in Patients with Infective Endocarditis

Jing Yong Ng[1,*], Eu Fon Tan[2], Soubhagyashree Roy[1], Sungha Cho[2], Takakazu Ryan Yatoji Tan[2], Marsioleda Kemberi[1] andWael I Awad[1,3,4]

*[1]Barts Heart Centre. St Bartholomew's Hospital, London, UK; [2]Barts and The London School of Medicine and Dentistry, Queen Mary University of London, UK; [3]William Harvey Research Institute, Queen Mary University of London, London, UK; [4]University of South Wales, Cardiff, UK*

**Supplementary Table 1. Subgroup analysis of those who underwent valvular repair/replacement vs those who had concomitant coronary artery bypass graft (CABG).**

| | **Valve** | | **Valve + CABG** | | **P value** |
|---|---|---|---|---|---|
| | **Total N=** | **Mortality (%)** | **Total N=** | **Mortality** | |
| Total | 385 | 48 (12.5%) | 53 | 9 (17.0%) | 0.3 |
| Normal | 185 | 10 (5.4%) | 13 | 2 (15.4%) | 0.7 |
| Moderate | 131 | 9 (6.9%) | 23 | 4 (17.4%) | 0.2 |
| Severe | 83 | 18 (21.7%) | 12 | 3 (25.0%) | 0.9 |
| Haemodialysis | 34 | 11 (32.4%) | 5 | 0 | 0.3 |

**Supplementary Table 2. Subgroup analysis of patients who had preoperative cardiogenic shock versus those who did not. P values in bold indicate statistical significance.**

| | **Preop Cardiogenic Shock** | | **No Preop Cardiogenic Shock** | | **P value** |
|---|---|---|---|---|---|
| | Total N= | Mortality (%) | Total N= | Mortality | |
| Total | 50 | 16 (32.0%) | 437 | 41 (9.4%) | <0.001 |
| Normal | 9 | 2 (22.2%) | 189 | 10 (5.3%) | 0.04 |
| Moderate | 12 | 3 (25.0%) | 142 | 10 (7.0%) | 0.03 |
| Severe | 16 | 6 (37.5%) | 80 | 15 (18.8%) | 0.1 |
| Haemodialysis | 13 | 5 (38.5%) | 26 | 6 (23.1%) | 0.3 |

**Supplementary Table 3. Subgroup analysis of patients who had preoperative atrial fibrillation (AF) versus those with sinus rhythm. P values in bold indicate statistical significance.**

| | **AF** | | | **Sinus Rhythm** | | | **P value** |
|---|---|---|---|---|---|---|---|
| | Total N= | Mortality (%) | | Total N= | Mortality | | |
| **Total** | 51 | 11 | 21.6% | 437 | 44 | 10.1% | 0.01 |
| **Normal** | 8 | 2 | 25.0% | 188 | 10 | 5.3% | 0.1 |
| **Moderate** | 17 | 3 | 17.6% | 134 | 9 | 6.7% | 0.3 |
| **Severe** | 20 | 6 | 30.0% | 73 | 15 | 20.5% | 0.6 |
| **Haemodialysis** | 6 | 0 | 0.0% | 30 | 10 | 33.3% | 0.2 |
